# Critical Issues in Psychological Services Within Italian Schools: Definition, Contexts, Interventions, and Legislation for Vulnerable Populations

**DOI:** 10.3390/children12040433

**Published:** 2025-03-29

**Authors:** Simona Grilli, Giulio Perrotta, Stefano Eleuteri, Irene Petruccelli

**Affiliations:** 1 Human and Social Sciences Department, Mercatorum Universitas, 00186 Rome, Italy; i.petruccelli@icloud.com; 2 Department of Clinical and Odontostomatological Sciences (DISCO), Faculty of Medicine and Surgery, Polytechnic University of Marche, 60020 Ancona, Italy; g.perrotta@pm.univpm.it; 3 Faculty of Medicine and Psychology, Sapienza University of Rome, 00185 Rome, Italy; stefano.eleuteri@uniroma1.it

**Keywords:** school psychologist, educational psychology, social psychology, COVID-19, mental health

## Abstract

Background: This paper presents a comprehensive literature review that investigates the pivotal role of school psychologists within the Italian educational context, highlighting their importance in promoting mental health and students’ well-being, emphasizing the importance of a comprehensive school psychology evidence-based service to facilitate the personal and professional development of both students and staff. Methods: To conduct this review, we utilized databases such as Scopus, ResearchGate, PsycINFO, and PubMed to access recent scientific literature related to school psychologists in Italy, focusing primarily on studies published between 2018 and 2025. We employed keywords including “school psychologist in Italy”, “mental health”, “COVID-19”, “COVID-19 and school psychologists”, and “school psychologist legislation in Italy” to guide our search. Results: By synthesizing the existing literature, this study aims to explore the integration and effectiveness of psychological services in Italian schools, particularly in light of the challenges posed by the COVID-19 pandemic. This review provides an overview of the current state of school psychology evidence-based services in Italy, with particular attention to the emotional and psychological challenges exacerbated by the pandemic. This event that has profoundly impacted community life highlighted the importance of mental health, prompting Italy to enhance the presence of psychologists within schools. Furthermore, this review critically analyzes the existing laws and psychological frameworks, drawing on data from an extensive examination of legislative documents and the previous literature regarding the implementation of school psychology services. The findings reveal that 69.2% of Italian schools adopted a school psychology service following legislative initiatives, aimed at addressing the trauma and discomfort caused by the pandemic. Conclusions: Despite this progress, the presence of this figure is still rare and while significant steps have been taken, a shift towards a more preventative and holistic model is currently essential.

## 1. Introduction

The school psychologist is a trained mental health professional who specializes in delivering psychological, educational, and behavioral support to students within the educational environment. This role is pivotal in fostering both the emotional well-being and academic success of students, necessitating collaboration with teachers, parents, and other professionals. The school psychologist not only addresses existing issues and discomforts but also plays a crucial preventive role in promoting overall well-being within the school setting. In this capacity, the psychologist acts as a catalyst for positive change, working synergistically with all stakeholders in the educational system [1,2]. The various professionals within the school—such as psychologists, educators, and pedagogues—do not compete with one another; rather, they are complementary and interdependent. Each professional, with their unique expertise, is expected to contribute collaboratively to enhance both the efficiency and effectiveness of the educational system. This collaborative approach also extends to preventive measures, emphasizing a shared vision of “caring” and a commitment to fostering a culture of teamwork that includes teachers, school personnel, and administrative staff [2,3]. In this context, it is essential to recognize that the school age is a particularly vulnerable period for students, as it is marked by significant developmental changes across multiple domains. During this stage, the risk of developing psychological issues peaks, especially among those exposed to stressful life events. Consequently, this evidence highlights the urgent need for targeted psychological support in educational settings for these at-risk populations [4,5,6]. The activities undertaken by the school psychologist are directed towards recognizing and addressing the diverse needs that arise within the school context, which should be understood not merely as a static institution but as a dynamic and multifaceted system. This system encompasses a variety of participants, including individuals (students, teachers, and school staff), groups (classes, educational teams, and families), and the various institutions with which the school interacts (families, the educational system, and community organizations). These elements are in constant interaction, influencing one another and contributing to the creation of a complex environment where individual and collective needs converge. Within this context, the school psychologist assumes the role of mediator and facilitator, engaging in interventions designed to support the system’s equilibrium and the relationships that define it [7].

## 2. Application Contexts and Specific Interventions

In the context of understanding the complexities surrounding psychological services within Italian schools, it is crucial to explore the various application contexts and specific evidence-based interventions that have been implemented. This examination provides a foundational understanding of how these services are tailored to meet the needs of vulnerable populations, setting the stage for subsequent discussions on the regulatory framework that governs these practices.

The demand for psychological interventions targeting children, families, teachers, and school staff is steadily increasing nowadays. Psychologists can play a significant role in various areas, both in preventative measures [8,9,10] and in implementing decisive interventions, whether on an individual basis or in group settings [11,12,13,14,15,16]. This is especially true for interventions aimed at reducing school dropout rates [14] or identifying and supporting students with special educational needs [17]. These needs may arise from clinical issues, such as specific learning disorders, attention deficit hyperactivity disorder, or mood and personality disorders, as well as from social challenges, including cultural and/or linguistic disadvantages or deteriorating social–family contexts that exacerbate relational conflicts. Interventions are critical for promoting the inclusiveness of children from immigrant families [18], as well as for the prevention and detection of deviant or antisocial behavior [19,20], such as bullying [21,22], and those resulting from psychological trauma [23,24] or specific psychiatric disorders [25,26,27,28].

To effectively address these critical issues, it is imperative that the school psychologist not only possesses specialized training but also engages in continuous professional development, as the dynamics within the school environment are perpetually evolving. It is essential for the school psychologist to collaborate with experts from various fields, such as the Postal Police in matters related to cyberbullying, or nutritionists concerning eating disorders. To navigate the complexities and diversity inherent in a school environment, it is essential to acquire a comprehensive set of skills that complement and transcend strictly professional competencies. These skills encompass a thorough understanding of the regulations governing educational operations, knowledge of ethical principles, and a heightened awareness of ethical considerations throughout various stages of one’s professional responsibilities [2,29]. In a study conducted by Popov and Spasenovic [30] regarding the number of students per psychologist in schools, a different picture emerges in different countries. Notably, the number of students per psychologist on average is around 250–500; the best pattern seems to appear in Croatia, where there are two professionals within schools with about 180 students, which becomes four in institutions with more than 500 students. In the United States, the average number of students per psychologist is 250, while in other countries, such as Russia and Bulgaria, the number of students amounts to 500 per school psychologist. In the Italian context, however, there is a clear criticality within the school system related to the absence of a stable and structured school psychology service, unlike the reality in other European countries. The Italian reality, on the other hand, is characterized by the presence of only one school psychologist for all students in the institution, even if these are complexes in which several levels exist—such as preschool, elementary school, and secondary school—thus reaching a very high total number of students. Italy is one of the few countries in which the presence of a psychologist in schools is not mandatory. Subsequently, it is not uncommon for this professional figure to be absent from school buildings entirely. To date, as previously noted, the Italian school system has mainly involved psychologists for interventions related to developmental difficulties, with a predominantly diagnostic approach, or to combat school dropout and social marginalization. These evidence-based interventions, which are often of limited duration, have generally stemmed from social-health initiatives promoted by Local Health Authorities. For example, the Information and Consultancy Centers (CIC) are regulated by Italian law no. 162 of 1990, which aims to provide psychological support for children and adolescents but does not enforce a consistent presence of psychologists in schools [31,32].

In recent years, two significant laws have been introduced to address the shortcomings in the Italian school psychology system. The first, Law No. 56/1989, established the framework for the professional practice of psychologists in Italy, emphasizing the need for licensed professionals in educational settings. However, the law lacks enforcement mechanisms, leading to inconsistencies in implementation across schools. The second, Law No. 3/2018, aims to improve mental health services, including in schools, by promoting more comprehensive access to psychological support. This law mandates that schools implement mental health programs and services, which could lead to a more structured and accessible psychology service within educational institutions. The existing literature presents numerous collaborations, projects, successes, advantages, and advancements concerning psychology in educational settings; however, these efforts remain insufficient to address such a significant need. Recently, several schools have initiated internal calls for tenders to procure psychological counselling services, utilizing both qualified internal staff and external professionals. In recent years, local authorities have endeavored to support schools by promoting lists of qualified professionals and initiatives related to “school psychology”. The primary issue with these initiatives lies in the limited availability of hours, which is often restricted to just 30 to 40 h per year. Additionally, some schools employ economic selection criteria that allocate extra points to professionals who provide their services at lower rates. For instance, there are remuneration offers as low as EUR 18 per hour [33,34,35]. In Italy, this service is provided only following a public call for applications, for medium to short periods and specific projects, with remuneration for the professional often significantly lower than the average for freelancing, thus disfavoring participation and aggregation. A health and educational policy more centered on this role and this service is needed in order to guarantee the constitutional right to mental health and a healthy workplace. It is important to clarify that a significant number of objectives and functions pertaining to the health and well-being of both children and adults can be identified in the literature, all closely linked to the school environment. Specifically, as for the student population, the research indicates that access to mental health services and support within schools has a direct impact on enhancing the physical and psychological safety of individuals, as well as their academic performance. Furthermore, this access yields positive outcomes for socio–emotional learning [36,37,38,39,40].

## 3. The Regulatory Aspects of Italian Legislation: Is a New and Decisive Legislative Proposal Needed?

Building upon the insights gained from the previous section regarding application contexts and interventions, it becomes evident that a robust regulatory framework is essential for supporting and enhancing the efficacy of psychological services in schools. This section will delve into the current legislative landscape in Italy, critically analyzing whether existing regulations adequately address the evolving needs of vulnerable populations or if a new legislative proposal is warranted to address the gaps and discrepancies.

Article 1 of the Italian Law no. 56 of 18 February 1989 describes the psychologist’s profession as one that ‘includes the use of cognitive and intervention tools for prevention, diagnosis, rehabilitation and support activities in the field of psychology addressed to the person, the group, social organisms and communities’. Within the Italian school context, the psychologist can therefore be seen as the professional who works with—and towards—individuals and groups belonging to the school institution and, therefore, to its community. According to the aforementioned law, moreover, the psychologist is the professional in charge of protecting the psychological health of the population; that is to say, he or she corresponds to a health professional—as is also inferred by Italian Law no. 3 of 11 January 2018, which configures psychology as a health profession, recognizing the relevance of psychological aspects for health—qualified to practice the profession and enrolled in the Professional Register of the Region in which he or she resides or works, as well as required to comply with the Deontological Code of Italian Psychologists. With reference precisely to the latter, according to what is stated in Art. 3 called the Principle of Responsibility, the psychologist has the ‘duty to increase knowledge about human behaviour and use it to promote the psychological well-being of the individual, the group and the community’ and ‘in every professional sphere they work to improve people’s ability to understand themselves and others and to behave in a conscious, congruous and effective manner, being aware of the fact that ‘in their professional practice they can intervene significantly in the lives of other people’. Further, Art. 11 states that ‘psychologists and psychologists are strictly bound to professional secrecy. Therefore, they shall not disclose news, facts or information learned by reason of their professional relationship’, as well as, according to Art. 9 on Informed Consent, being obliged in their activity ‘to adequately inform the persons involved with respect to the purposes, procedures, methods, times and risks of the same, as well as the manner of processing of personal data collected in order to acquire their consent’. No less important, Art. 5 on Professional Competence requires psychologists to ‘maintain an adequate level of preparation and professional updating, with particular regard to the fields in which they work’, i.e., ongoing training necessary to ensure quality and efficiency of the service (Presidential Decree No. 137 of 7 August 2012). Currently in the Italian context, schools, thanks to the didactic and organizational autonomy of individual institutions (Law no. 59 of 15 March 1997) and the so-called ‘Good School’ (Law no. 107 of 13 July 2015), can make use of the assistance of a psychologist through direct agreements with the professionals themselves, with local health authorities, with regional school offices, with students and their families, as well as by resolution of the collegiate bodies, using contributions from institutions, banking institutions, parents or associations, or, drawing from the Institute Fund. Unfortunately, the absence of a clear regulatory framework generates a problematic scenario, whereby psychological activities in schools are not only performed by psychologists—as established by national legislation—but also by other figures who often lack the necessary skills for such a role. It follows that the repercussions of such negligence are manifested not only within the professional sector, but especially towards the users of ‘psychological’ services, i.e., those who, usually unwittingly, turn to operators who do not have the appropriate qualifications to ensure the protection of the mental health and psychological well-being of the individual and the community. Suffice it to consider, in this regard, the figure of the ‘counsellor’, of whom one hears more and more frequently nowadays, and who, moreover, is sometimes enrolled in lists created by associations and not in Orders recognized at the national legislative level, thus generating confusion and misunderstandings. The activity performed by this figure often tends to overlap inappropriately with that of the psychologist. This happens when the boundaries between the two professions are not clearly delineated, giving rise to situations in which the specific skills and responsibilities of each professional are mixed. For this reason, a recent ruling by the Lazio Regional Administrative Tribunal sanctioned the need to establish a clear distinction between the two figures, in order to protect the quality of services offered to users, ensuring that people receive appropriate assistance from professionals who are adequately trained and recognized in their field of competence. This is Judgement No. 13,020 of 17 November 2015, which, in addressing the issue of the distinction between the professional figures of the psychologist and the counsellor, clearly states that counselling activities cannot replace those of a psychologist, and that counsellors cannot practice in areas reserved for psychologists. Bearing in mind the directives provided in Art. 3 of the Italian Psychologists’ Code of Ethics on the Principle of Responsibility and the recent revision, of 25 September 2023, of Art. 31 of the same, concerning informed health consent, which places both adults and minors or incapacitated persons at the center of the decision-making process concerning psychological health treatment (giving relevance to the minor or incapacitated person, who must be listened to, and his or her wishes taken into account according to his or her age and maturity). Considering, in addition, that within the school environment most users are minors, it is essential that psychological interventions in schools are directed and managed precisely by qualified psychologists. To continue with the discussion of the prescriptions established by the Code of Ethics, art. 5 on Professional Competence establishes that the psychologist and the psychologist “recognise the limits of their competence and therefore use only theoretical-practical tools for which they have acquired adequate competence and, where necessary, formal authorisation” and “employ methodologies for which they are able to indicate the sources and scientific references and do not raise unfounded expectations in the client and/or user”, while art. 6 on Professional Autonomy indicates that “in collaboration with professionals from other disciplines, the psychologist and the psychologist exercise full professional autonomy while respecting the other’s competence”, recalling the importance of cooperation with other professionals and the importance of the professional autonomy of the psychologist and the psychologist, wishing to recall the importance of interdisciplinary cooperation while maintaining their decisional and operational independence, but at the same time, recognizing and respecting the specific knowledge and the value of the contribution of each professional figure, avoiding interfering or overlapping in the areas of competence of those who practice another profession, thus operating within the limits of their own discipline.

In Italy, the issue of psychological support in schools has seen, over time, a series of interventions that have approached the issue from different perspectives, with specific tools and methods. These evidence-based approaches have had different purposes, depending on the context and emerging needs, with the aim of responding to the challenges related to students’ psychological well-being and to the integration between the educational dimension and emotional support. The current legislation on the figure of the school psychologist is evolving. Currently, there is a legislative proposal (no. 520)—presented on 7 November 2022 and discussed for the first time in the VII Culture Commission of Montecitorio on 14 December 2023—that provides for the introduction of the figure of the psychologist in all schools of all grades. The discussion of the aforementioned bill is expected to reach Parliament this year, and while waiting for the bill to be approved, the Minister of Education and Merit signed a three-year protocol with the National Council of the Order of Psychologists, aimed at supporting the school system through initiatives aimed at both preventing psychological distress and promoting the well-being of students. Italian draft law no. 520, filed in the Chamber of Deputies under the first signature of congresswoman Carmen di Lauro, aims to establish the figure of the professional psychologist on a structural and permanent basis in Italian schools, thus giving value and relevance to the psychophysical well-being of students and school staff. Wanting to go into the specifics of the content of the legislative proposal on the establishment of the professional figure of the school psychologist, we wish to reiterate, first of all, the purpose of the contribution of this role, namely the promotion of well-being, the prevention of work-related stress, as well as phenomena such as school drop-out or addictions, the fight against bullying, and the improvement in relational dynamics. The proposal is integrated with existing measures, such as “the psychological bonus”, an Italian initiative that aims to offer additional support to young individuals during their formative and developmental stages, enabling them to access psychological counselling services at no cost. Should it be approved, it will, in fact, represent a real revolution for the school world. The text of the bill sets out the functions to be performed by this professional figure, which are listed as follows: it establishes a supportive relationship with pupils and students on an individual and group basis; it may participate, when deemed necessary, in the teachers’ board, class councils, and parents’ receptions and act as an advisor to the school head, also with a view to embarking on interdisciplinary paths or participating in educational projects. With reference to the work performed by the school psychologist, the following areas of evidence-based interventions are reported: support in the insertion, or reintegration, following periods away from home; supporting the construction of students’ personalities and the development of emotional and social life skills; provision of an empowering and motivating learning environment; supporting the well-being of students and school staff; early detection of situations of deviance, such as bullying and cyberbullying, and distress, such as eating disorders and addictions, as well as special educational needs; support and training for teachers regarding specific developmental age issues and any relational difficulties existing within the classroom between teachers and students; support and training for teaching staff and administrative, technical, and auxiliary staff for the better management of difficult situations; psychological counselling for families to support parenting; interaction, where needed, with other professionals working in various capacities within the school; implementation of peer support projects, as well as projects aimed at promoting and fostering relationships between peers, improving social skills and fostering support within groups and social formations; individual and group psychological counselling for students, teaching and auxiliary staff, and parents, aimed at optimizing school performance and human relations, supporting the student’s training and growth process, preventing discomfort, pathologies and deviance, and enhancing parental responsibilities in school training. The school psychologist provides the class councils and the board of teachers with all useful elements for improving the relational dynamic, personalizing the educational offer and assessing the pupils or students; the school psychologist, on the instructions of the head teacher, may organize interviews with the family and any other person he or she considers relevant to the development of the student; the school psychologist has access to all information held by the school. Finally, with regard to the necessary requirements, those who (1) hold a master’s degree in developmental psychology, (2) are duly registered in the professional register of psychologists, and (3) have at least three years’ professional experience in educational contexts are eligible for the position of school psychologist.

In summary, should this bill be approved, the impact of this law on schools would be profound. It would not only institutionalize the presence of school psychologists but also fundamentally transform the way schools address mental health and well-being.

## 4. Before and After COVID-19: The Lesson “Perhaps” Learned at Great Cost

### 4.1. The Psychological Impact of the COVID-19 Pandemic and Legislative Responses in Italy

As the discussion shifts from the regulatory considerations presented in the previous section, it is essential to acknowledge the substantial changes instigated by the COVID-19 pandemic. This pandemic profoundly affected various facets of community life, leading to a radical transformation of everyday experiences for individuals.

As far as psychology in general is concerned, the effects and revolutions that have ensued are innumerable, including, certainly, the importance that mental health has assumed in this particular historical period on a global level, leading psychology to downgrade certain approaches and focus on new challenges related to the management of psychological well-being in such a crisis context. It is now well known and documented in the literature how the pandemic has had serious repercussions on the psychological health of individuals and communities, increasing and amplifying problems related to experiences of anxiety, depression, and stress-related disorders. Aspects such as social isolation, fear of contagion, and general perceived uncertainty formed the basis for the co-construction of a psychologically challenging and hostile environment for many people, especially for those categories characterized by greater vulnerability such as children, adolescents, the elderly, or those already suffering from pre-existing mental disorders. In addition, elements such as feelings of loneliness and alienation were severely and continuously amplified by the quarantine, the lack of direct social support, and the need to adapt to new ways of living, such as remote work, distance learning, or safe distance. Following the pandemic, however, the Italian Ministry of Education signed a Memorandum of Understanding with the National Council of the Order of Psychologists on 16 October 2020. This protocol aims to provide psychological support to school staff, students, and families in all Italian schools—from kindergarten to secondary school—for the 2020–2021 school year, with the objective of responding to the trauma and discomfort caused by the COVID-19 emergency and, at the same time, preventing the onset of psychophysical disorders. As a consequence of this legislative intervention, 69.2% of Italian schools (which corresponds to 5662 of 8183 institutes) have implemented the school psychology service, while some of these schools have strengthened their already existing system of psychological counters; for many others (3178/5662 institutes), it was the first time. A recent systematic review [41] examined the studies that have assessed the mental health of children and adolescents during the first year of the COVID-19 pandemic. Most of these studies have reported an increase in depressive and anxious symptoms among participants, as well as a general deterioration in mental health compared to the period preceding the pandemic. Restriction of social interactions, due to government control policies, was associated with more depressive and anxious symptoms, particularly among older children, adolescents, and girls. Specifically, neurodiverse children and adolescents, as well as those with pre-existing mental illnesses, have experienced higher levels of psychological distress, depression, anxiety, and behavioral problems since the start of the pandemic. Excessive exposure to and use of the Internet and social media have been correlated with the mental distress experienced by children and adolescents [42,43,44,45]. To counteract these repercussions, the promotion of positive mental well-being can have important impacts on developing resilience and helping to prevent negative mental health outcomes [46]. Activities such as exercise, access to entertainment, positive family relationships, and social support have been shown to be correlated with better mental health outcomes in numerous studies [47,48,49], suggesting that they could make an important contribution to the maturation of mental resilience during the pandemic. It should also be mentioned that protective health behaviors for children and adolescents, which have been drastically reduced or suppressed by the pandemic [44,46,50]—such as sufficient movement, routine, sleep, and nutrition—are positively associated with social and emotional health for both children and adolescents [51] and could be important targets for interventions. Particularly in relation to social support, it has been demonstrated that such support is significantly associated with a reduction in symptoms of depression, anxiety, and insomnia. Given the importance of social connectedness as a fundamental determinant of mental well-being in children and adolescents [52,53], they require access to meaningful social support, especially in the context of diminished opportunities for face-to-face interaction [41].

### 4.2. Adapting School Psychology for a Post-Pandemic Reality: From Crisis Intervention to Preventative Care

In this context, it is not surprising that the significance of school psychology has increased markedly as the pandemic progressed. This is attributable to the emergence of new needs and requirements among students, as well as the exacerbation of existing challenges. The complex and challenging circumstances arising from the health emergency related to COVID-19 have underscored the critical importance of having psychological and social services that are not only adequately structured but also well-organized and highly effective. These services must be capable of responding promptly and competently to the various challenges posed by the crisis, encompassing both health and social dimensions, while offering concrete and targeted solutions to the emerging needs of the population. In a context marked by significant collective stress, the necessity for a support system capable of swiftly adapting to emerging needs has become evident, particularly within the educational environment. Here, both students and teachers have had to contend not only with the suspension of face-to-face teaching but also with the psychological distress associated with remote learning, isolation from peers, and uncertainty regarding their educational and social futures. It is often observed that following a crisis, there is an acceleration in addressing needs that have previously been overlooked or disregarded. This is notably true in the case of the school psychologist. The primary issue that requires clarification pertains to the target population for their interventions [54]. More specifically, the dichotomy presents, on one hand, an approach focused on the management of discomfort, and on the other, an effort primarily directed towards the promotion of well-being. Bombi and colleagues [55] assert that the school is a ‘privileged observatory’ for the early detection of signs of adjustment or maladjustment in students, enabling the timely implementation of interventions that can mitigate the impact of any difficulties, thereby preventing potential future problems. It is fundamental, therefore, that the school psychologist can work not only on discomfort, but also on the promotion of well-being and psychophysical health, intervening in a preventative manner. Unfortunately, however, the scarcity of resources and available hours often leads to focus only on crisis situations, reinforcing, at the cultural level, a vision of psychology that intervenes only in conditions of malaise. What is suggested is an approach that goes beyond the mere management of difficulties, and instead includes prevention programs aimed at strengthening relational, environmental, and personality protective factors, diminishing the influence of risk factors and facilitating the development of more favorable developmental paths. The psychologist’s intervention should be ‘ecological’, i.e., aiming to improve the adaptation between the individual and his environment. His or her action must be characterized by versatility and adaptability to the various contexts and developmental stages of the students—from children to adolescents—and with different ways of involving teachers and parents, bearing in mind that the specific needs of a given school, as well as its social and cultural background, must be taken into account. The main objective of the school psychologist’s work should correspond to the activation of skills and resources that are already present in the school system, but which are latent. In this sense, the psychologist is configured as a “bridge” between the various systems of reference, required not only to possess a solid and widespread store of knowledge, but also to be able to adapt it effectively to the specific context, characterizing school psychology as a mediating discipline and not as an “allology” [54].

### 4.3. Mental Health of Children and Adolescents During COVID-19

Italy has been one of the countries most significantly impacted by the pandemic, both within Europe and globally. According to data from the World Health Organization (WHO), from 3 January 2020 to 16 July 2021, there have been a total of 4,278,319 confirmed cases and 127,840 reported deaths. For this reason, the closure of educational institutions has lasted far longer than originally planned, resulting, as a natural consequence, in a drastic and unforeseen reduction in social interactions for students and subjecting them to a significant challenge to their well-being. Pre-adolescents, who are at a stage characterized by significant physical, psychological, and social changes due to the onset of puberty, have been particularly affected by the repercussions of this crisis. This developmental phase is critical for the mental health and overall development of young people, rendering it vulnerable to potential psychological issues. If these issues are not identified and addressed in a timely manner, they may escalate into more serious disorders. One such concern is social anxiety disorder, which frequently manifests during adolescence, although it can also emerge earlier. Thus, early detection is essential for effective prevention. Experiencing social anxiety—regardless of whether a formal diagnosis has been made—can lead to a variety of negative outcomes, including feelings of loneliness, diminished enjoyment of school, an increased reliance on maladaptive coping strategies, and subpar academic performance [56]. The prevention of this disorder seems to assume particular relevance in the Italian context, since the recent literature has revealed high levels of social anxiety experienced by Italian adolescents [57]. In addition, the pre-adolescent phase is characterized by an increase in relationships between peers, especially in the value attributed to them, both in terms of sentimental and friendship relationships, and maintaining a good level of such relationships constitutes an important index of psychological well-being, as well as a protective factor for young people in the onset of various disorders [58]. It is not surprising, therefore, that the limitation of opportunities for contact with peers due to lockdown, both in the classroom and outside, has had a serious impact on children’s health. In this respect, on the other hand, to the youngest children, i.e., those attending primary school, especially those with certified neurodevelopmental disorders, the main problems are highlighted at the level of school learning, but also regarding the experience of anxiety and depressive states. The latter are aggravated by the fact that minors, at this early age, have greater difficulty in recognizing and verbalizing their emotional experiences, thus tending more easily to express their discomforts in the form of somatic manifestations. Another significant issue that has arisen because of the pandemic pertains to the increased amount of time that young individuals spent online and on social networks. This includes activities such as playing video games and engaging in chat and video chat with friends and acquaintances.

These behaviors had a clear impact on the alteration of students’ daily routines and their circadian rhythms with regard to the organization of the sleep-wake cycle, with consequences on both the quantity and quality of sleep and thus affecting aspects such as the reduction in impulse control and attentional capacity [59], as well as implying negative effects on immune functions [60].

### 4.4. Reflections from a School Psychologist’s Perspective

At this point, we would like to examine an interesting contribution that offers an overview of the Italian situation following COVID-19, from the point of view of a personal experience lived by a school psychologist [61]. The author offers clinical considerations, deriving from her experience, with the aim of contributing practical implications for the psychological service in Italian schools. In particular, it has emerged that the students proved to be enthusiastic and willing to open up and confide in a professional figure, without the fear of being judged or made fun of by their peers. In fact, even though there were not many hours available, the students, informed of this limitation, were very willing to participate and request psychological support. The author reports having perceived a sense of urgency by many of the young people, who, from the very first meetings, showed great openness and initiative in recounting their experiences, memories, dreams, doubts, and hopes, as if they were looking forward to this listening space dedicated to them. The way students proceeded to provide information, getting straight to the point, without getting lost in pleasantries, with remarkable clarity and order in the presentation of events, impressed the psychologist, who, at the end of the sessions, felt that a great weight had been lifted. The logistical constraints imposed by the limited time also seemed to have had a positive effect, as they enabled the pupils to confide in each other more quickly, proving that psychological intervention in schools, even if reduced in terms of time, proves to be very useful and effective. What the psychologist felt was that for the pupils, it was not so much a matter of ’doing something’ or intervening in response to specific discomforts—activities that would naturally require a considerable amount of time—but rather the opportunity to be heard without judgement. It was essential for them to have the presence of a mental health professional who could engage with them emotionally and empathetically, creating an environment in which they felt comfortable expressing their thoughts, dreams, and fears. Contrary to what usually happens in private practice where it is the parents who seek first contact, the author reports having received in this case numerous requests from the students themselves, demonstrating, once again, the strong motivation and willingness of these children—especially pre-adolescents—to be able to tell their stories. However, even in cases where the first approach was initiated not on the students’ initiative, but at the suggestion of parents or teachers, the students in question immediately showed interest and had no difficulty opening up. This last observation mainly concerns the youngest children, i.e., those attending primary school, who, for obvious reasons, were introduced to the service by their parents. They expressed a high level of satisfaction with the initial meeting, to the extent that they indicated a willingness to participate again in the future. It is evident that parents and teachers play a crucial role in facilitating students’ access to psychological support, particularly when students are either unaware of such services or reluctant to seek help due to concerns about their peers’ reactions. In this context, adults can assist in taking the initial steps towards accessing these essential services. It is noteworthy to highlight that when students sought assistance from the psychological service, they not only did not experience any ridicule from their peers, but, in fact, their classmates were more inclined to approach the psychologist themselves. This represents a significant advancement in the perception of psychologists, viewing them as a valuable resource rather than as adversaries or as medical professionals to be consulted in secrecy. This shift aids in reducing the potential stigma associated with seeking psychological counselling among peers and encourages students to be more willing to make appointments. In this regard, it is assumed that the pandemic has contributed to the process of de-stigmatization and awareness of mental illness and, consequently, to the increasing acceptance of seeking psychological help. It is no coincidence that between March 2020 and February 2022, 67.2% of psychologists reported an increase in requests compared to the pre COVID-19 period, particularly from adolescents (81%), who mainly reported anxiety and depressive symptoms, relationship problems, self-harming behaviors, and eating disorders [62]. Not only problems and critical issues, however, because, as noted by the author, many students used the sessions to talk about friendships, romantic relationships, passions, and dreams, highlighting, again, the emerging need of pre-adolescents to have the opportunity to be listened to by someone who is willing to do so and who is not a relative, teacher, or friend. Facilitating access to psychological support in schools during this crucial and formative stage of development for young individuals may encourage them to be more receptive to seeking psychological assistance in the future, should the need arise. The psychologist’s experience reported by Loscalzo [61] offered the students not only the opportunity to tell their stories and be listened to, but identified that other communication channels, such as drawing or music, were also used. Some pre-adolescents, in fact, were better able to convey their emotions and feelings and to establish a relationship more easily precisely through these alternative artistic means. Their use was usually advanced precisely on the initiative of the students themselves, demonstrating the fact that incorporating such tools in school psychology practice can foster the construction of a deeper relationship and connection and involve the students more in psychological reflection on themselves. To conclude, a further element that proved effective, particularly in working with immigrants, was the adoption of English to communicate with those who had difficulty expressing themselves in Italian. In fact, although it may seem difficult or troublesome to use a non-native language to conduct a more rational conversation, the author asserts that based on her personal experience, it was possible to establish a deep connection and conduct an emotional conversation in this mode as well. This is due to the fact that empathy is mainly conveyed through body language, while words are only a tool to initiate dialogue and create a relationship. Suffice it to say that just feeling free to cry in front of someone willing to take care of you professionally, without moral judgments on your feelings, thoughts, or actions, is able to ease the pain of a difficult life lived in loneliness (perhaps in a new country where the integration process has not yet been completed and you still do not have solid points of reference). In fact, the effectiveness of psychological support is found even if the psychologist does not understand every single word or if the person cannot fully express his or her feelings in their native language. Therefore, the author recommends—in the case of international students—the use of English (or any other non-native language as a possible meeting point with people) to all professionals who have knowledge of it, in order to prevent language from becoming an obstacle both in the work and in the previous phase of the student’s request (who might be blocked precisely by a language barrier). When both the student and the psychologist are communicating in a non-native language, the student may feel less judged for his or her limited language skills and working together to understand each other in English (or another non-native language) may foster and strengthen the relationship building.

Taking a cue from the aforementioned article, we would also like to emphasize the importance of maintaining continuity in the school psychology desk service, possibly ensuring that the same psychologist or psychologist continues to operate within the same school. This is for several reasons [62,63]: the professional in question would have the opportunity to acquire in-depth knowledge of school dynamics, both those of an organizational nature and those inherent to the relational sphere, with obvious advantages for their future work and interventions in the years to come; the users of the psychological service—both students and teachers—having built up a relationship of trust with the psychologist during the first year, could benefit from continuity in the support received, keeping the same contact person. This would avoid them having to start from scratch with a new professional, facilitating a more stable and consistent path of assistance, which would also be particularly advantageous for those who need prolonged support over time; if the school psychologist were included among the school staff, they could guarantee their presence and availability during all teaching hours, and not only during those few limited hours per week. Their full-time presence, in fact, would contribute to improving the quality of the service offered, allowing the psychologist to be available on demand for timely or group interventions in the classroom—that are also frequently requested by teachers. Finally, it would be desirable to have psychologists of different genders within the same school complex, so that students could be offered the possibility of choosing the therapist with whom they feel most comfortable. This option would be particularly relevant for people from cultural backgrounds where gender differences are considered a sensitive element within interpersonal relationships. Providing students with the opportunity to make choices may foster a relationship of trust and enhance the effectiveness of psychological support.

The first systematic nationwide research initiative targeted on gaining an understanding of the profession suggested that school psychology in Italy has followed a distinct evolution which has developed thus far into refined forms of consultation that are strongly correlated with relational ties between school psychologists and their schools [64]. More recent articles suggested some future developments for school psychologists in Italy, where individual counseling to students is predominant and a school-based psychological helpdesk is the most common form of provision of psychological services. It emerges the need to promote a new policy design and provide more structured evidence-based psychological services in schools [65,66].

## 5. Conclusions

The findings of this review article underscore the pivotal role of school psychologists in addressing the complex psychological needs within educational settings. Their evidence-based contributions extend beyond crisis management, as they are essential in crafting comprehensive support systems that foster positive growth and development among students. As recognized by Italian Law no. 3 of 11 January 2018, psychology is configured as a health profession, underscoring the importance of psychological aspects for overall health. The role of school psychologists involves welcoming and addressing needs within the dynamic context of schools, understood as articulated systems where individual and collective needs intersect. They serve as mediators and facilitators of growth processes through interventions aimed at supporting systemic balance and relationships. This includes efforts to reduce school dropout rates, support students with special educational needs, address specific clinical or social challenges such as learning disorders or cultural disadvantages [20,21], prevent deviant behaviors like bullying [23,24], and manage psychological trauma or psychiatric disorders [25,26,27,28,29]. Despite the increasing demand for psychological interventions targeting children, families, teachers, and staff, several limitations hinder the effectiveness of school psychologists in Italy. Notably, the absence of mandatory psychologist presence in schools contributes to a fragmented approach to mental health support. Legislative proposals, such as Proposal no. 520 for mandatory psychologists across all educational levels, reflect the growing recognition of mental health’s significance within schools. However, these proposals have not yet been fully realized, leaving many schools without adequate psychological resources. Additionally, while initiatives like the Memorandum between the Ministry of Education and the National Council of Psychologists have led to significant implementation (69.2%) of school psychology services during the 2020–2021 year, challenges persist regarding resource availability. Many school psychologists are primarily perceived as crisis responders, which limits their ability to adopt proactive, preventive strategies. This reactive perception can undermine the potential for early identification of distress signs and the implementation of early intervention strategies. A crucial limitation of this study is the reliance on the existing literature that may not fully capture the diverse experiences and challenges faced by school psychologists across different contexts. Future research should explore the experiences of school psychologists more comprehensively, including qualitative studies that delve into their roles, challenges, and perceptions within various educational settings. Additionally, longitudinal studies could assess the long-term impacts of school psychology interventions on student outcomes. In conclusion, integrating school psychologists into Italy’s educational system is crucial for enhancing students’ mental health outcomes. Their collaboration with educators, families, and communities can help shape an inclusive landscape that prioritizes the well-being of all students. By addressing the limitations identified in this discussion, future efforts can further strengthen the role of school psychologists, ensuring that they are equipped to implement both preventive and responsive strategies tailored to the diverse needs of the student population.

## References

[1] Bulgarelli D., Chiaruttini P., Gavello C., Massia S., Muscatello V., Spinelli S., Silvia R.V. (2019). Lo Psicologo Scolastico. Cenni Normativi e Buone Prassi.

[2] Matteucci M.C. (2018). Psicologi scolastici: Quale formazione in Italia. Psicol. Dell’educazione.

[3] Cornoldi C., Molinari L. (2019). Lo Psicologo Scolastico. Competenze e Aree di Intervento.

[4] Kessler R.C., Berglund P., Demler O., Jin R., Merikangas K.R., Walters E.E. (2005). Lifetime prevalence and age-of-onset distributions of DSM-IV disorders in the National Comorbidity Survey Replication. Arch. Gen. Psychiatry.

[5] Matucci G. (2021). La scuola nell’emergenza pandemica, fra inclusione e solidarietà. Quad. Cost..

[6] Sisk L.M., Gee D.G. (2022). Stress and adolescence: Vulnerability and opportunity during a sensitive window of development. Curr. Opin. Psychol..

[7] Petter G. (2016). Lo Psicologo Della Scuola.

[8] Zurma S. (2023). Prospettive di Psicologia Scolastica.

[9] Durlak J.A. (2015). Handbook of Social and Emotional Learning: Research and Practice.

[10] Keng S.L., Smoski M.J., Robins C.J. (2011). Effects of mindfulness on psychological health: A review of empirical studies. Clin. Psychol. Rev..

[11] Rigby K. (2012). Bullying in schools: Addressing desires, not only behaviours. Educ. Psychol. Rev..

[12] Haugland B.S.M., Haaland A.T., Baste V., Bjaastad J.F., Hoffart A., Rapee R.M., Wergeland G.J. (2020). Effectiveness of brief and standard school-based cognitive-behavioral interventions for adolescents with anxiety: A randomized noninferiority study. J. Am. Acad. Child Adolesc. Psychiatry.

[13] Miller D.N., Eckert T.L., Mazza J.J. (2009). Suicide prevention programs in the schools: A review and public health perspective. Sch. Psychol. Rev..

[14] Kourkoutas E.E., Xavier M.R. (2010). Counseling children at risk in a resilient contextual perspective: A paradigmatic shift of school psychologists’ role in inclusive education. Procedia-Soc. Behav. Sci..

[15] Fabiano G.A., Evans S.W. (2019). Introduction to the special issue of School Mental Health on best practices in effective multi-tiered intervention frameworks. Sch. Ment. Health.

[16] Gobat N., Littlecott H., Williams A., McEwan K., Stanton H., Robling M., Evans R. (2021). Developing a whole-school mental health and wellbeing intervention through pragmatic formative process evaluation: A case-study of innovative local practice within the School Health Research network. BMC Public Health.

[17] Caprara G.V., Barbaranelli C., Pastorelli C., Bandura A., Zimbardo P.G. (2000). Prosocial foundations of children’s academic achievement. Psychol. Sci..

[18] Buscema L., Caridà R., De Luca G. (2024). Lineamenti di Legislazione Scolastica per L’inclusione.

[19] Dovigo F., Pedone F. (2019). I Bisogni Educativi Speciali. Una Guida Critica per Insegnanti.

[20] Cirese M. (2020). Minori Migranti. Diritti e Tutela dei Legami Familiari.

[21] Perrotta G., Marciano A. (2022). The clinical boundary between deviant behavior and criminal conduct: From maladaptive positions to pathological dysfunctionality using the “Graded Antisociality Model” (GA-M), the “Antisocial Severity Scale” (AS-S) and “Perrotta-Marciano questionnaire on the grade of awareness of one’s deviant and criminal behaviors” (ADCB-Q). Ann. Psychiatry Treat..

[22] Piccininno D., Perrotta G. (2024). The Analysis of Criminogenic Factors in a Sample of Drug Addicts: The Relevance of the Social -Environmental Element in Antisocial Behavior. Pilot Study. Open J. Trauma.

[23] Olweus D. (2013). School bullying: Development and some important challenges. Annu. Rev. Clin. Psychol..

[24] Fraguas D., Díaz-Caneja C.M., Ayora M., Durán-Cutilla M., Abregú-Crespo R., Ezquiaga-Bravo I., Arango C. (2021). Assessment of School Anti-Bullying Interventions: A Meta-analysis of Randomized Clinical Trials. JAMA Pediatr..

[25] Perrotta G. (2020). Psychological trauma: Definition, clinical contexts, neural correlations and therapeutic approaches. Curr. Res. Psychiatry Brain Disord..

[26] Perrotta G., Fabiano G., Posta F. (2023). The psychopathological evolution of “Behavior and Conduct Disorder in Childhood”: Deviant and criminal traits in preadolescence and adolescence. A review. Open J. Pediatr. Child Health.

[27] Perrotta G. (2022). The new Dysfunctional Personality Model of the Anxiety Matrix (DPM-AM): “Neurotic Personality Disorder” (NPD). Ann. Psychiatry Treat..

[28] Perrotta G. (2024). The new Dysfunctional Personality Model of the Dramatic Matrix (DPM-DM) and the Psychotic Matrix (DPM-PM): “Dramatic Personality Disorder” (DPD) and “Psychotic Personality Disorder” (PPD). Psychol. Ment. Health Care.

[29] Giberti F., Rossi R. (2023). Manuale di Psichiatria.

[30] Matteucci M.C. (2023). Lo Psicologo Scolastico. Aree e Strumenti per L’intervento.

[31] Popov N., Spasenovic V. (2020). School Counseling: A Comparative Study in 12 Countries. Bulg. Comp. Educ. Soc..

[32] Mittino F. (2023). Raccontare, Raccontarsi. Lo Spazio D’ascolto Psicologico in Ambito Scolastico.

[33] Amendolia A.S., Psicologo scolastico (2019). Una review sul ruolo professionale, sul confronto con gli altri paesi UE e sulla situazione in Italia. In I Quaderni della Fondazione degli Psicologi della Toscana; Tuscany, Italy. https://www.fondazionepsicologi.it/wp-content/uploads/2020/01/Psicologo-scolastico_.pdf.

[34] Zullig K.J., Huebner E.S., Patton J.M. (2011). Relationships among school climate domains and school satisfaction. Psychol. Sch..

[35] Huebner E.S., Diener C. (2008). Research on life satisfaction of children and youth. The Science of Subjective Well-Being.

[36] Meroni C., Fagnani L., Confalonieri E., Baventore D., Velasco V. (2021). The Italian School Psychologists’ Role: A Qualitative Study about Professional Practices and Representations. Eur. J. Investig. Health Psychol. Educ..

[37] Matteucci M., Coyne J. (2017). School Psychology in Italy: Current status and challenges for future development. ISPA World Go Round.

[38] Eklund K., DeMarchena S.L., Rossen E., Izumi J.T., Vaillancourt K., Rader Kelly S. (2020). Examining the role of school psychologists as providers of mental and behavioral health services. Psychol. Sch..

[39] Saulle R., De Sario M., Bena A., Capra P., Culasso M., Davoli M., Minozzi S. (2022). School closures and mental health, wellbeing and health behaviours among children and adolescents during the second COVID-19 wave: A systematic review of the literature. Epidemiol. Prev..

[40] Hawrilenko M., Kroshus E., Tandon P., Christakis D. (2021). The association between school closures and child mental health during COVID-19. JAMA Netw. Open.

[41] Samji H., Wu J., Ladak A., Vossen C., Stewart E., Dove N., Snell G. (2022). Mental health impacts of the COVID-19 pandemic on children and youth–a systematic review. Child Adolesc. Ment. Health.

[42] Ma L., Mazidi, Li M., Li K., Li Y., Chen S., Kirwan R., Wang Y. (2021). Prevalence of mental health problems among children and adolescents during the COVID-19 pandemic: A systematic review and meta-analysis. J. Affect. Disord..

[43] Oswald T.K., Rumbold A.R., Kedzior S.G., Moore V.M. (2020). Psychological impacts of “screen time” and “green time” for children and adolescents: A systematic scoping review. PLoS ONE.

[44] Moore S.A., Faulkner G., Rhodes R.E., Brussoni M., Chulak-Bozzer T., Ferguson L.J., Tremblay M.S. (2020). Impact of the COVID-19 virus outbreak on movement and play behaviours of Canadian children and youth: A national survey. Int. J. Behav. Nutr. Phys. Act..

[45] Pecoraro L., Dalle Carbonare L., De Franceschi L., Piacentini G., Pietrobelli A. (2020). The psychophysical impact that COVID-19 has on children must not be underestimated. Acta Paediatr..

[46] Keyes C.L. (2007). Promoting and protecting mental health as flourishing: A complementary strategy for improving national mental health. Am. Psychol..

[47] Yang C., Gao H., Li Y., Wang E., Wang N., Wang Q. (2022). Analyzing the role of family support, coping strategies and social support in improving the mental health of students: Evidence from post COVID-19. Front. Psychol..

[48] Duan H., Yan L., Ding X., Gan Y., Kohn N., Wu J. (2020). Impact of the COVID-19 pandemic on mental health in the general Chinese population: Changes, predictors and psychosocial correlates. Psychiatry Res..

[49] Gadermann A.C., Thomson K.C., Richardson C.G., Gagné M., McAuliffe C., Hirani S., Jenkins E. (2021). Examining the impacts of the COVID-19 pandemic on family mental health in Canada: Findings from a national cross-sectional study. BMJ Open.

[50] Liu Y., Zhang E., Li H., Ge X., Hu F., Cai Y., Xiang M. (2024). Physical activity, recreational screen time, and depressive symptoms among Chinese children and adolescents: A three-wave cross-lagged study during the COVID-19 pandemic. Child Adolesc. Psychiatry Ment. Health.

[51] Rollo S., Antsygina O., Tremblay M.S. (2020). The whole day matters: Understanding 24-hour movement guideline adherence and relationships with health indicators across the lifespan. J. Sport Health Sci..

[52] Jose P.E., Ryan N., Pryor J. (2012). Does social connectedness promote a greater sense of well-being in adolescence over time?. J. Res. Adolesc..

[53] Shochet I.M., Dadds M.R., Ham D., Montague R. (2006). School connectedness is an underemphasized parameter in adolescent mental health: Results of a community prediction study. J. Clin. Child Adolesc. Psychol..

[54] Toso C. (2021). Uno psicologo tra i banchi di scuola. Psicol. Clin. Dello Svilupp..

[55] Bombi A.S., Bucciarelli M., Cornoldi C., Menesini E. (2014). Perché la Scuola non può fare a meno della Psicologia (e invece qualche volta se ne dimentica)?. G. Ital. Psicol..

[56] Weeks M., Coplan R.J., Kingsbury A. (2009). The correlates and consequences of early appearing social anxiety in young children. J. Anxiety Disord..

[57] Giannini M., Loscalzo Y. (2016). Social anxiety and adolescence: Interpretation bias in an Italian Sample. Scand. J. Psychol..

[58] Hartup W.W., Stevens N. (1997). Friendships and adaptation in the life course. Psychol. Bull..

[59] McLaughlin-Crabtree V., Witcher L.A., Ivanenko A. (2008). Impact of sleep loss on children and adolescents. Sleep and Psychiatric Disorders in Children and Adolescents.

[60] Gozal L., Gozal D. (2008). The multiple challenges of obstructive sleep apnea in children: Diagnosis. Curr. Opin. Pediatr..

[61] Loscalzo Y. (2022). Psychological counseling during the COVID-19 pandemic: Clinical thoughts and implications arisen from an experience in Italian schools. Int. J. Environ. Res. Public Health.

[62] Ciardi L. La Nazione. Serve Aiuto Dagli Psicologi. Il COVID fa volare le Richieste [We Need Help from Psychologists. The COVID Makes Requests Fly]. https://www.lanazione.it/firenze/cronaca/serve-aiuto-dagli-psicologi-il-covid-fa-volare-le-richieste-1.7715167.

[63] Liu S.M., Oakland T. (2016). The Emergence and Evolution of School Psychology Literature: A Scientometric Analysis from 1907 Through 2014. Sch. Psychol. Q..

[64] Trombetta C., Alessandri G., Coyne J. (2008). Italian School Psychology as Perceived by Italian School Psychologists: The Results of a National Survey. Sch. Psychol. Int..

[65] Matteucci M.C., Farrell P.T. (2018). School psychologists in the Italian education system: A mixed-methods study of a district in northern Italy. Int. J. Sch. Educ. Psychol..

[66] Matteucci M.C., Soncini A., Floris F., Truscott S.D. (2025). School psychology in Italy: A mixed-method study of actual and desired roles and functions. Sch. Psychol. Int..

